# Supplementary material for: The Utility of Bedside Assessment Tools and Associated Factors to Avoid Antibiotic Overuse in an Urban PICU of a Diarrheal Disease Hospital in Bangladesh
Source: Antibiotics (Basel). 2021 Oct 15;10(10):1255. doi: 10.3390/antibiotics10101255 (PMC8532929; doi:10.3390/antibiotics10101255)
Supplement: Supplementary file 1 [file antibiotics-10-01255-s001.zip › antibiotics-1364705-supplementary.pdf]

S1 File. Pediatric Intensive Care Unit admission criteria

Admit to ICU if has any 1 major or 3 minor criteria:

Major

- Severe sepsis/ Septic Shock
- Requires respiratory support:
  - Invasive: (mechanical ventilation in respiratory failure)
  - Non-invasive: (bubble CPAP in hypoxemia and grunting respiration)
- Convulsion/Disorientation/Coma
- Hypothermia <35.5^{o} Celsius
- Hypoglycemia (≥2 times)

Minor

- SAM or WAZ score <-4
- Restlessness/Lethargy
- Hyperthermia ≥ 40° Celsius (core temperature)
- Moderate to severe neutropenia (moderate <1.0 to ≥0.5×10^{9}/L; severe <0.5×10^{9}/L)
- Thrombocytopenia <100,000
